# Progressively Inpainting Images Based on a Forked-Then-Fused Decoder Network

**DOI:** 10.3390/s21196336

**Published:** 2021-09-22

**Authors:** Shuai Yang, Rong Huang, Fang Han

**Affiliations:** 1 College of Information Science and Technology, Donghua University, Shanghai 201620, China; shuai.yang@mail.dhu.edu.cn (S.Y.); yadiahan@dhu.edu.cn (F.H.); 2 Engineering Research Center of Digitized Textile & Apparel Technology, Ministry of Education, Donghua University, Shanghai 201620, China

**Keywords:** image inpainting, contextual attention, feature fusion, multi-stage

## Abstract

Image inpainting aims to fill in corrupted regions with visually realistic and semantically plausible contents. In this paper, we propose a progressive image inpainting method, which is based on a forked-then-fused decoder network. A unit called PC-RN, which is the combination of partial convolution and region normalization, serves as the basic component to construct inpainting network. The PC-RN unit can extract useful features from the valid surroundings and can suppress incompleteness-caused interference at the same time. The forked-then-fused decoder network consists of a local reception branch, a long-range attention branch, and a squeeze-and-excitation-based fusing module. Two multi-scale contextual attention modules are deployed into the long-range attention branch for adaptively borrowing features from distant spatial positions. Progressive inpainting strategy allows the attention modules to use the previously filled region to reduce the risk of allocating wrong attention. We conduct extensive experiments on three benchmark databases: Places2, Paris StreetView, and CelebA. Qualitative and quantitative results show that the proposed inpainting model is superior to state-of-the-art works. Moreover, we perform ablation studies to reveal the functionality of each module for the image inpainting task.

## 1. Introduction

Image inpainting, which has been a research hotspot in the computer vision community, aims to fill in corrupted regions of an image with visually realistic and semantically plausible contents [1]. Its applications include photo-editing [2,3,4], computer-aided relic restoration [5,6,7,8], de-occlusion [9,10], privacy protection [11,12,13], aesthetic assessment [14], and virtual try-on systems [15,16]. The ill-posedness of image inpainting can be distilled into the following: how to seek the most proper hypothesis for the corrupted region conditioned on the valid surroundings. In the past decade, researchers have devoted substantial efforts to this field, which can be mainly divided into three categories: diffusion-based methods [17,18,19,20], patch-based methods [21,22,23,24,25,26], and CNN (Convolutional Neural Network)-based methods [27,28,29,30,31,32,33,34,35,36,37,38,39,40,41,42,43,44,45,46,47,48,49,50].

Based on the priori knowledge that image pixels are piece-wise smooth, the diffusion-based methods [17,18,19,20] establish a variety of anisotropic PDEs (Partial Differential Equations) for modeling the process of information diffusion. Although these methods attempt to mimic the paradigm of manual inpainting, they are suitable only for the corrupted region with slender shape and homogeneous texture.

The patch-based methods [21,22,23,24,25,26], which exploit the non-local self-similarity of images, typically operate through the following steps: feature extraction, similarity calculation, candidate screening, and texture synthesis. Unfortunately, these methods focus only on the low-level features and fail to perceive the overall semantics of a given image. It is virtually impossible for the patch-based methods to create semantically meaningful contents, so that they usually suffer setbacks when dealing with the task of face completion.

Nowadays, we are witnessing all-round breakthroughs in the computer vision community, mainly caused by the deep CNNs and the powerful large-scale parallel computing devices (e.g., the graphics processing unit). In general, the CNNs are constructed as a hierarchical architecture with depth, which is conducive to capturing rich features geared towards a specific task. More interestingly, some exquisite networks, such as GAN (Generative Adversarial Network) [51] or VAE (Variational Auto-Encoder) [52], excel at creating realistic samples. Thus, the CNN-based methods [27,28,29,30,31,32,33,34,35,36,37,38,39,40,41,42,43,44,45,46,47,48,49,50] have been a recent surge of interests in the field of image inpainting. Pathak et al. [27] set up a CE (Context Encoder) network that is of a channel-wise fully connected layer in the middle. Remarkably, an adversarial mechanism, which is similar in spirit to GAN, is introduced into the learning procedure for generating visually clear fillings. Using dual discriminators, Iizuka et al. [28] developed a variant of the CE network [27], which encourages global coherence and local consistency. Yang et al. [29] proposed a multi-scale neural patch synthesis approach, in which a tailor-made loss function was designed to guide the procedures of preserving contextual structures and of generating fine-grained contents. However, although these methods have the capability to create novel contents, there still exist semantic faults or visual artifacts such as color discrepancy and texture distortion. To address these problems, advanced inpainting mechanisms in the following three aspects: specialized convolution operations [30,31,32,33], contextual attention module [34,35,36,37,38,39,40,41,42,43,44,45], and progressive inpainting strategies [46,47,48,49,50], have been studied recently.

In the first aspect, Liu et al. [30] proposed a partial CNN, which is specialized to the task of image inpainting. Therein, each partial convolution kernel acts only on the valid pixels and thus effectively resist the interference derived from the corrupted regions. After each partial convolutional layer, a vanilla rule-based operation is triggered to update the masked region with the goal of shrinking its area layer by layer. In [31], the partial convolution kernel was used to process structure and texture features, which produces multiple feature streams with different scales. Yu et al. [32] put forward a learnable dynamic feature selection mechanism, which can be viewed as a generalized version of the partial convolution. Specifically, a group of accompanying convolution kernels are configured at each layer for learning channel-wise soft-gating masks. Using the element-wise multiplication, the learned masks modulate the feature maps for adaptively filtering out the interference. Ma et al. [33] devised region-wise convolutions and deployed them into the decoder network. As its name implies, the region-wise convolution is to separate the tasks of reconstructing the valid region and inferring the corrupted region.

In the second aspect, Song et al. [34] designed a contextual attention module based on patch-swap operation. Unlike the traditional patch-based methods, their proposal not only takes feature maps as surrogates for texture propagation but also embeds the contextual attention module seamlessly into the entire learnable network. Yan et al. [35] inserted a shift-connection layer into the decoder network, which explicitly borrows contextual information from encoder’s feature map. Yu et al. [36] computed attention scores over feature maps through the convolution operations and normalized these scores by applying the softmax function. During network training, feature patches in the contextual region are weighted by the normalized attention scores for texture synthesis. This attention module [36] was adopted in a multi-task inpainting framework [37] for processing multi-modal features extracted from image, edge and gradient maps. Sagong et al. [38,39] constructed a shared encoder network for reducing the number of convolution operations and modified the means of computing the attention scores. Uddin and Jung [40] designed a global-and-local attention module and aimed to refine the inpainting-oriented features by integrating global dependency and local similarity information. In this attention module, an effective mask pruning mechanism was developed to filter out features with interference. More recently, Yu’s contextual attention module [36] has been extended to a multi-scale version in [41], to a cascaded version in [42], to a pyramid version in [43], to a locally coherent version in [44], and to a knowledge consistent version in [45].

In the third aspect, Xiong et al. [46] explicitly separated the whole inpainting task into three parts in sequence to perceive image foreground, to complete object contour, and to fill in corrupted region. Zhang et al. [47] proposed a progressive generative neural network for semantically inpainting images. Inspired by the concept of curriculum learning, they added a LSTM (Long Short-Term Memory) component [53] into the middle of U-net [54] to store and share the inpainting knowledge between multiple stages. Guo et al. [48] invented a full-resolution residual block, which learns to inpaint a local region covered by one dilation. Stacking such blocks in series helps to progressively fill in the corrupted region. Unfortunately, this method can only deal with small holes, i.e., the area up to 96 pixels in diameter. Chen and Hu [49] progressively completed the image inpainting task from the perspective of pyramid multi-resolution, in which lower-resolution inpainting is followed by higher-resolution inpainting iteratively. Zeng et al. [50] proposed to evaluate predications’ confidence during the progressive process of inpainting. The confident regions, which serve as feedback information, were encouraged to cover as large corruption as possible.

However, there still exist some problems in these advanced inpainting mechanisms. First, although the partial convolution can restrict itself to absorb information from the valid region, the frequently used fully spatial feature normalization may still introduce interference. Second, feature patches lying inside the corrupted region usually contain larger deviations. This phenomenon misleads the contextual attention module and incurs wrong attention allocation. Third, the progressive inpainting strategies, in general, employ the learnable convolution kernels to perceive the periphery of the corrupted region but neglect the contextual information outside the receptive field.

To alleviate these problems, in this paper, we propose a novel end-to-end multi-stage pipeline mainly consisting of a shared encoder network and a forked-then-fused decoder network. The encoder network aims to capture the useful information from the valid region and to block out the objectionable interference derived from the corrupted region. To this end, we design a new network unit, called PC-RN, which equips the partial convolutional layer [30] with the region-wise feature normalization [55]. The decoder network, at the beginning, forks into two branches, called local reception branch and long-range attention branch, respectively. To ensure local consistency, the former is responsible for perceiving the valid information and for reconstructing the local field around the corrupted region. To generate fine-grained details, the latter resorts to two cascaded MSCA (Multi-Scale Contextual Attention) modules, both of which basically follow the attention mechanism in [41], for flexibly borrowing features from remote spatial positions. Two feature flows are then adaptively refined through a SE (squeeze-and-excitation)-based [56] fusing module.

Our proposal is to fill in the whole corrupted region progressively. Each inpainting stage only targets a limited area of the corrupted region, thereby somewhat alleviating the problem of wrong attention allocation. Furthermore, thanks to the SE-based fusing module, each inpainting stage can comprehensively utilize the local and long-range features extracted by the double branches.

We conducted extensive experiments and comparative studies on three benchmark databases: Places2 [57], Paris StreetView [58], and CelebA [59]. To support the above claims, we visualize the feature deviations within the corrupted region and exhibit how each region contributes to the inpainting performance across the multiple stages. Additionally, qualitative and quantitative results demonstrate the effectiveness and the superiority of the proposed model compared with state-of-the-art works.

The rest of the paper is organized as follows. Section 2 gives a detailed description of the proposed model. Section 3 introduces the experimental programs and exhibits the corresponding results. Section 4 summarizes this paper and draws some conclusions.

## 2. Our Model

**Figure 1:**
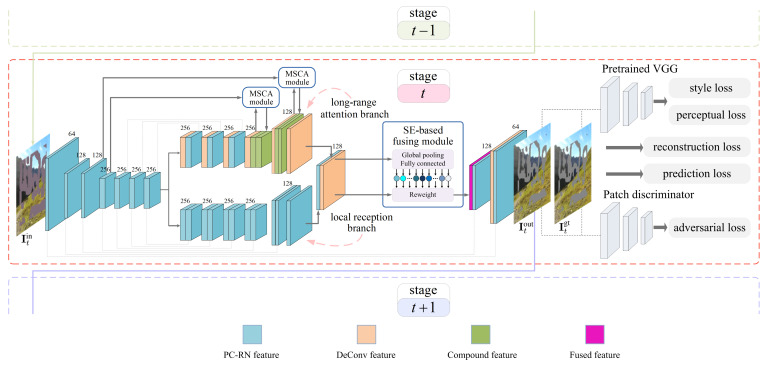
The overall architecture of the proposed inpainting network. Details of the MSCA module and the SE-based fusing module are illustrated schematically in Figure 2 and Figure 3, respectively.

The overall architecture of the proposed inpainting network is schematically illustrated in Figure 1. Let $I_{t}^{\mathrm{in}}$, $I_{t}^{\mathrm{out}}$, and $I_{t}^{\mathrm{gt}}$ denote the input, output, and groundtruth images, respectively, at the tth inpainting stage. The shared encoder network captures the useful information from the valid region of $I_{t}^{\mathrm{in}}$. Then, the resulting feature map is fed into the forked-then-fused decoder network for image generation, yielding $I_{t}^{\mathrm{out}}$. Comparing $I_{t}^{\mathrm{out}}$ with $I_{t}^{\mathrm{gt}}$, we calculate various losses: reconstruction loss, perceptual loss, style loss, and adversarial loss, with the aid of a pre-trained VGG (Visual Geometry Group) network [60] and a patch-based discriminator network [61]. We collectively use these losses to guide the end-to-end training. The inpainting network at the tth inpainting stage restricts its attention to a limited area of the corrupted region. Its output image $I_{t}^{\mathrm{out}}$ acts as the input image for the next inpainting stage, namely that $I_{t+1}^{\mathrm{in}}=I_{t}^{\mathrm{out}}$. Without loss of generality, we elaborate on a single inpainting stage hereafter. The subscript *t* is dropped for clarity, unless explicitly needed to distinguish between multiple inpainting stages.

### 2.1. Shared Encoder Network

Unlike generic computer vision tasks, which process full information, the image inpainting task is to deal with incomplete information. Hence, how to resist the incompleteness-caused interference becomes a critical issue for the inpainting network, especially for the shallow layers. To cope with this issue, in this paper, we combine the partial convolution [30] and the region normalization [55] and take them as a basic unit, called PC-RN, to construct the inpainting network. The PC-RN unit provides an elegant way, which is immune against the interference, to process the incomplete information and paves the way for generating high-quality results. Hereafter, we give a brief introduction to the PC-RN unit.

We define $X\in R^{C\times H\times W}$ as an input feature map of a PC-RN unit, where *C*, *H*, and *W* represent the number of channels, height, and width, respectively. Let M denote a binary mask of size H×W, which takes value 0 inside the corrupted region and 1 elsewhere. Suppose that a partial convolution kernel of size C×S×S currently encompasses a local part, denoted by x, of the input feature map X. Correspondingly, we use m to represent the local binary mask that is covered by the kernel. Let w and b denote the weights and biases of the kernel, respectively. Mathematically, the current partial convolution, which yields a response $x^{\prime }$, can be expressed as

(1) $$x^{\prime }=\left\{\begin{array}{cc} w^{T}\left(x⊙m\right)\frac{S^{2}}{\mathrm{sum}(m)}+b, & \mathrm{if}\;\;\mathrm{sum}(m)>0,\\ 0, & \mathrm{otherwise}, \end{array}\right.$$

where ⊙ denotes Hadamard product while sum(m) counts the number of 1s in m. The scaling factor $S^{2}/\mathrm{sum}\left(m\right)$ makes an appropriate compensation for the corrupted positions because they are absent from the calculation course of Equation (1). After each partial convolution, the local binary mask m is updated as follows:

(2) $$m^{\prime }=\left\{\begin{array}{cc} 1, & \mathrm{if}\;\;\mathrm{sum}(m)>0,\\ 0, & \mathrm{otherwise}. \end{array}\right.$$

The convolved feature map, denoted by $X^{\prime }$, and the updated binary mask, denoted by $M^{\prime }$, are composed of $x^{\prime }$ and $m^{\prime }$, respectively. We denote the sizes of $X^{\prime }$ and $M^{\prime }$ by $C^{\prime }\times H^{\prime }\times W^{\prime }$ and $H^{\prime }\times W^{\prime }$, respectively.

Feature normalization acts to standardize the mean and variance of the convolved feature map for stabilizing learning. In our proposal, such normalization is performed in a region-wise fashion. Specifically, we first properly resize M to the resolution of $M^{\prime }$, namely $H^{\prime }\times W^{\prime }$. Then, according to M (the resized version) and $M^{\prime }$, the feature map $X^{\prime }$ is partitioned into three regions, namely the valid region $R^{V}$, the filled region $R^{F}$, and the corrupted region $R^{C}$. Their formal definitions are as follows:

(3) $$\begin{array}{cc} R^{V} & =\left\{\left(i,j\right)|m\left(i,j\right)=1\;\;\mathrm{and}\;\;m^{\prime }\left(i,j\right)=1\right\},\\ R^{F} & =\left\{\left(i,j\right)|m\left(i,j\right)=0\;\;\mathrm{and}\;\;m^{\prime }\left(i,j\right)=1\right\},\\ R^{C} & =\left\{\left(i,j\right)|m\left(i,j\right)=0\;\;\mathrm{and}\;\;m^{\prime }\left(i,j\right)=0\right\}, \end{array}$$

where (i,j) represents a spatial coordinate with $1⩽i⩽H^{\prime }$ and $1⩽j⩽W^{\prime }$. Note that $R^{C}$ may become *⌀* after several PC-RN units (and several inpainting stages), meaning that all of the corrupted positions have been assigned by predictions. For each region, calculate its mean and standard deviation as follows:

(4) $$\mu_{k}^{U}=\frac{1}{|R^{U}|}\sum_{\left(i,j\right)\in R^{U}}x^{\prime }\left(k,i,j\right),$$

(5) $$\sigma_{k}^{U}=\sqrt{\frac{1}{|R^{U}|}\sum_{\left(i,j\right)\in R^{U}}{\left(x^{\prime }\left(k,i,j\right)-\mu_{k}^{U}\right)}^{2}+\epsilon },$$

where U={V,F,C} and $|R^{U}|$ stands for the cardinality of the set $R^{U}$. The subscript *k*, of which the value lies in the interval $\left[1,C^{\prime }\right]$, is the index of a channel. The notation ϵ is a prescribed small constant for numerical stability. The region-wise feature normalization can be formulated as

(6) $$x^{\prime \prime }\left(k,i,j\right)=\frac{1}{\sigma_{k}^{U}}\left(x^{\prime }\left(k,i,j\right)-\mu_{k}^{U}\right).$$

Finally, region-wise affine transformations based on a set of learnable parameters $\left\{\gamma_{k}^{U},\beta_{k}^{U}\right\}$, where U={V,F,C}, are separately applied to the normalized feature values.

As shown in Figure 1, we set up the shared encoder network by cascading seven PC-RN units. Throughout our proposal, the partial convolution kernel is of size 3×3 and has “same” mode for zero-padding. Downsampled convolution is realized by setting the stride to 2.

### 2.2. Forked-Then-Fused Decoder Network

The decoder network, which receives the output feature map of the encoder network, forks into the local reception branch and the long-range attention branch. Then, a SE-based fusing module adaptively refines the feature maps from the two branches. In addition to the main body of the network, extra skip connections, which concatenate two feature maps as shown in Figure 1, are added to avoid information loss during the forward pass and to mitigate the vanishing gradient problem during the backward pass.

#### 2.2.1. Local Reception Branch

The local reception branch is expected to infer the corrupted region conditioned on the valid surroundings. In the early inpainting stages, however, the shared encoder network may fail to cover the entire corrupted region. In other words, the input to the decoder network still contains the corrupted region, namely $|R^{C}|>0$. To prevent the interference, the PC-RN unit is reused here for constructing the six-layer local reception branch, as shown in Figure 1. The upsampled convolution is realized by setting the stride to 1/2. In the later inpainting stages, $|R^{C}|$ eventually becomes 0. Under this circumstance, the partial convolution and the region normalization naturally degenerate into the standard convolution and batch normalization, respectively.

#### 2.2.2. Long-Range Attention Branch

The long-range attention branch, of which the core component is the MSCA module, aims to infer the corrupted region by borrowing features from distant spatial positions. In particular, the standard convolutions, rather than the partial convolutions, are used in this branch, with the goal of making a rough prediction for the whole corrupted region. The MSCA module operates on a pair of feature maps, denoted by $X_{n^{-}}$ and $Y_{n^{+}}$. The former $X_{n^{-}}$ represents the feature map at the nth-to-end layer of the shared encoder network, while the latter $Y_{n^{+}}$ is the one generated by the nth convolutional layer of the long-range attention branch.

First, we combine $X_{n^{-}}$ with $Y_{n^{+}}$ via the following form

(7) $${\tilde{Y}}_{n}=X_{n^{-}}⊙M_{n}+Y_{n^{+}}⊙\left(1-M_{n}\right),$$

where $M_{n}$, taking value 0 inside the filled region and 1 elsewhere, denotes the binary mask accompanied with $X_{n^{-}}$ and $Y_{n^{+}}$. Since the standard convolution fills in the whole corrupted region at a time, the resulting feature map contains only two kinds of regions, namely the valid region $R^{V}$ and the filled region $R^{F}$.

**Figure 2:**
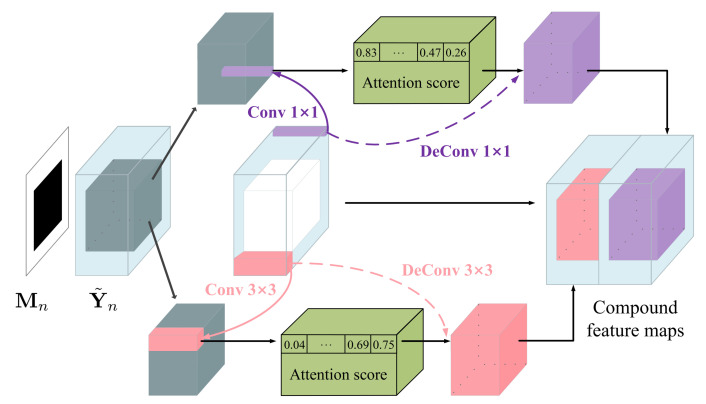
The schematic illustration of the multi-scale contextual attention (MSCA) module.

Second, as shown in Figure 2, we divide ${\tilde{Y}}_{n}$ into multi-scale patches of size 1×1 and 3×3 and compute the inter-patch normalized inner product

(8) $$a\left(i^{V},j^{V},i^{F},j^{F}\right)=\langle \frac{{\tilde{y}}_{n}\left(i^{V},j^{V}\right)}{\|{\tilde{y}}_{n}\left(i^{V},j^{V}\right)\|},\frac{{\tilde{y}}_{n}\left(i^{F},j^{F}\right)}{\|{\tilde{y}}_{n}\left(i^{F},j^{F}\right)\|}\rangle ,$$

where ${\tilde{y}}_{n}\left(i^{V},j^{V}\right)$ and ${\tilde{y}}_{n}\left(i^{F},j^{F}\right)$ represent the patches of ${\tilde{Y}}_{n}$ centered at $\left(i^{V},j^{V}\right)$ in the valid region and centered at $\left(i^{F},j^{F}\right)$ in the filled region, respectively. It is worth mentioning that Equation (8) can be effectively implemented using convolution, in which ${\tilde{y}}_{n}\left(i^{V},j^{V}\right)$ serves as the kernel. We then use the softmax function to exponentiate and normalize the inter-patch similarity along the $i^{V}$-$j^{V}$ dimension. The processed result, denoted by $a^{☆}\left(i^{V},j^{V},i^{F},j^{F}\right)$, is called the attention score.

Third, we reuse ${\tilde{y}}_{n}\left(i^{V},j^{V}\right)$ as the kernel and apply deconvolution to the attention score map. Such an inverse operation reconstructs the filled region, in the sense of integrating the valid patches through a weighted average way.

Finally, the filled region of ${\tilde{Y}}_{n}$ is replaced by the reconstructed counterpart, yielding a new compound feature map. Inspired by [41], we also consider the multi-scale scenario, where the patch sizes are 1×1 and 3×3, so that the MSCA module produces two compound feature maps, as shown in Figure 2. We concatenate $Y_{n^{+}}$ and the two compound feature maps to form the output of the $n^{\mathrm{th}}$ layer of the long-range attention branch.

Alternatively, we can propagate the attention scores over a small neighboring region along the horizontal and vertical directions. Mathematically, the horizontal version can be formulated as

(9) $$\hat{a}\left(i^{V},j^{V},i^{F},j^{F}\right)=\sum_{p=-b}^{b}a^{☆}\left(i^{V}+p,j^{V},i^{F}+p,j^{F}\right),$$

where *p* denotes a shift lying in the interval [−b,b]. Analogously, the vertical version imposes the shift *p* on $j^{V}$ and $j^{F}$, respectively. This trick is helpful because the neighboring region usually shares similar attention scores, and its effectiveness has been validated by [36].

It is worth noting the differences between the MSCA module and the multi-scale attention module used in [41]. First, a SE block [56] is configured in the original attention module [41] for refining the compound feature maps. By contrast, we move the SE block [56] to the fusing module (see the next section) for comprehensively refining the local and long-range features. Second, the original attention module [41] only processes the decoding feature map in a single-stage regime. Contrastively, we not only cascade two MSCA modules together for hierarchically synthesizing the inpainting-oriented features but also perform the MSCA modules in multiple inpainting stages. Consequently, for different stages, the MSCA module has different sources for synthesizing features. See the results of the ablation study in Section 3.4.4.

#### 2.2.3. SE-Based Fusing Module

Let $Z\in R^{C\times H\times W}$ denote a concatenation of the two feature maps obtained from the double branches. The SE-based fusing module takes Z as input.

**Figure 3:**
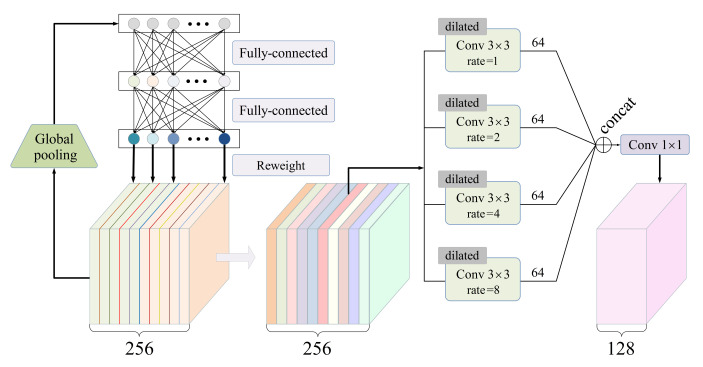
The schematic illustration of the SE-based fusing module, where the number of channels *C* is set to 256.

As shown in Figure 3, the SE (Squeeze-and-Excitation) [56] block processes Z through the following steps. First, the squeeze step applies an average-pooling operation to each channel of Z, with the goal of extracting a global feature vector with *C* elements. Second, the modulation step learns to properly transform the global feature vector into *C* weighting coefficients through a two-layer fully connected net. Third, the excitation step multiplies each channel of Z by the corresponding weighting coefficient.

Furthermore, four dilated convolutional layers with the same kernel size of C×3×3, in parallel, perceive the weighted feature map. Four dilation rates are set to 1, 2, 4, and 8. This ASPP (Atrous Spatial Pyramid Pooling)-like architecture [62] allows us to capture rich features from multi-scale receptive fields. Finally, a standard convolutional layer with a kernel size of C×1×1 is responsible for compressing a feature map by halving the number of channels. The SE-based fusing module outputs a feature map with the size C/2×H×W.

Note that, driven by data, all of the parameters in the SE-based fusing module are learnable and are jointly optimized together with other part of the network. Hence, this module has the capability to comprehensively refine the local and long-range features, making them more suitable for the image inpainting task.

### 2.3. Progressive Inpainting Strategy

The proposed network fulfills the image inpainting task in a progressive fashion, and each inpainting stage is in charge of inferring a limited area of the corrupted region by using the fused features.

Two binary masks, which share the same resolution as the input image $I_{t}^{\mathrm{in}}$, determines the to-be-filled region at the tth inpainting stage. The first one, denoted by $M_{t}^{\mathrm{in}}$, is called input binary mask. It takes value 0s for the corrupted region and 1s for the valid region. The second one $M_{t}^{\mathrm{out}}$, called output binary mask, stems from the last PC-RN unit of the shared encoder network. Here, proper upsampling is required for $M_{t}^{\mathrm{out}}$ to ensure the consistency of resolution. According to the update rule in Equation (2), we know that $M_{t}^{\mathrm{out}}$ takes 1s not only for the valid region but also for the filled region. Consequently, the to-be-filled region at the tth inpainting stage can be represented by $M_{t}^{\mathrm{out}}-M_{t}^{\mathrm{in}}$.

Let $I^{\mathrm{out}}$ and $I^{\mathrm{gt}}$ denote the output image of the inpainting network and the groundtruth image, respectively. At the tth inpainting stage, the resulting image $I_{t}^{\mathrm{out}}$ (or the groundtruth image $I_{t}^{\mathrm{gt}}$) is defined as $I^{\mathrm{out}}⊙M_{t}^{\mathrm{out}}$ (or $I^{\mathrm{gt}}⊙M_{t}^{\mathrm{out}}$). Furthermore, the inpainted results at the tth stage will be inherited by the next stage, in the sense that $I_{t+1}^{\mathrm{in}}=I_{t}^{\mathrm{out}}$ and $M_{t+1}^{\mathrm{in}}=M_{t}^{\mathrm{out}}$. The total number of inpainting stages *T*, which is a hyper-parameter, controls the trade-off between the inpainting quality and the computational cost. We stipulate that the output binary mask $M_{T}^{\mathrm{out}}$ is an all one-valued matrix, meaning that the corrupted region must be filled at the final inpainting stage.

The progressive inpainting strategy manages to fill in the central part of the corrupted region at the last few stages, with the aid of the inpainted results inherited from the previous stages. In other words, the progressive inpainting strategy allows the MSCA module to borrow features not only from the valid region but also from the filled region to alleviate the problem of wrong attention allocation. These claims are corroborated by the visualized results in Section 3.4.4.

### 2.4. Loss Function

In this paper, reconstruction loss, prediction loss, perceptual loss, style loss, and adversarial loss are collectively used to guide the network training.

The reconstruction loss measures the average error between $I_{t}^{\mathrm{out}}$ and $I_{t}^{\mathrm{gt}}$ at the pixel level. Its definition is

(10) $$L_{t}^{\mathrm{rec}}=\frac{1}{\mathrm{sum}(M_{t}^{\mathrm{out}})}∥I_{t}^{\mathrm{out}}-I_{t}^{\mathrm{gt}}{∥}_{1},$$

where ${∥\cdot ∥}_{1}$ denotes the $\ell_{1}$-norm of the enclosed argument.

The prediction loss, which focuses in particular on the filled region, measures the average error between the predicted pixel values and the groundtruth ones. Its definition takes the following form

(11) $$L_{t}^{\mathrm{pred}}=\frac{∥\left(M_{t}^{\mathrm{out}}-M_{t}^{\mathrm{in}}\right)⊙\left(I_{t}^{\mathrm{out}}-I_{t}^{\mathrm{gt}}\right){∥}_{1}}{\mathrm{sum}(M_{t}^{\mathrm{out}}-M_{t}^{\mathrm{in}})}.$$

The perceptual loss evaluates the inpainting quality at the semantic level. VGG19 network [60] pre-trained on the ImageNet database [63] is employed to extract the semantic features. Suppose that $\Phi_{t,l}^{U}$ is the lth extracted feature map for a given image $I_{t}^{U}$, where U={gt,out}. The size of $\Phi_{t,l}^{U}$ is denoted by $C_{l}\times H_{l}\times W_{l}$. With these preparations, the perceptual loss can be written as

(12) $$L_{t}^{\mathrm{percept}}=\sum_{l=1}^{L}\frac{∥M_{t,l}^{\mathrm{out}}⊙\left(\Phi_{t,l}^{\mathrm{out}}-\Phi_{t,l}^{\mathrm{gt}}\right){∥}_{1}}{\mathrm{sum}\left(M_{t,l}^{\mathrm{out}}\right)\cdot C_{l}},$$

where $M_{t,l}^{\mathrm{out}}$ that is of size $1\times H_{l}\times W_{l}$ refers to a downsampled version of $M_{t}^{\mathrm{out}}$. In this paper, we consider L=3 feature maps selected from the 4th, the 9th, and the 16th VGG19’s convolutional layers.

Gram matrix, which expresses the correlation between channels, can be viewed as a style indicator for a given image. We define a style loss, based on the Gram matrix, to evaluate the matching degree between two images. The Gram matrix is calculated as follows:

(13) $$\Psi_{t,l}^{U}=\frac{\left(M_{t,l}^{\mathrm{out}}⊙\Phi_{t,l}^{U}\right)\circ {\left(M_{t,l}^{\mathrm{out}}⊙\Phi_{t,l}^{U}\right)}^{T}}{\mathrm{sum}\left(M_{t,l}^{\mathrm{out}}\right)\cdot C_{l}},$$

where U={gt,out}. In Equation (13), the notation ∘ refers to a compound operation. It first reshapes its operands into matrices of size $C_{l}\times \left(H_{l}W_{l}\right)$ and then performs a matrix multiplication between the reshaped operands, yielding a $C_{l}\times C_{l}$ Gram matrix. Furthermore, the style loss is defined by

(14) $$L_{t}^{\mathrm{style}}=\sum_{l=1}^{L}\frac{1}{C_{l}^{2}}∥\Psi_{t,l}^{\mathrm{out}}-\Psi_{t,l}^{\mathrm{gt}}{∥}_{1}.$$

The adversarial loss quantifies the inpainting verisimilitude, with the aid of a patch-level discriminator network, as shown in Figure 1. In practice, the adversarial loss can be equivalent to a summation of two binary cross-entropy losses. That is

(15) $$L_{t}^{\mathrm{adv}}=\min_{D}\max_{G}\;L^{\mathrm{bce}}\left[D(I_{t}^{\mathrm{gt}}),1\right]+L^{\mathrm{bce}}\left[D(G\left(I_{t}^{\mathrm{in}}\right)),0\right],$$

where *D* and *G* stand for the patch-level discriminator network and the inpainting network, respectively. The bold symbol 1 (and 0) is a patch-level label matrix in which the elements are one-valued (and zero-valued). Each element in 1 (and 0) means that the corresponding feature patch is “real” (and “fake”). The loss function $L^{\mathrm{bce}}\left[a,b\right]$ computes the binary cross-entropy between a and b. Mathematically, its formula takes the following form:

(16) $$L^{\mathrm{bce}}\left[a,b\right]=\sum_{p=1}^{P}-\left[b_{p}\cdot \log\;a_{p}+\left(1-b_{p}\right)\cdot \log\;\left(1-a_{p}\right)\right],$$

where $a_{p}$ (and $b_{p}$) is the pth element of a (and b). The adversarial loss turns the network training into a min–max optimization problem, in which *G* and *D* collaborate each other and adapt to evolve together. Spectral normalization technique [64] is used to stabilize the training of the discriminator network.

In summary, the total loss used to guide the training of the entire network is as follows:

(17) $$L^{\mathrm{total}}=\sum_{t=1}^{T}\left(\lambda_{1}L_{t}^{\mathrm{rec}}+\lambda_{2}L_{t}^{\mathrm{pred}}+\lambda_{3}L_{t}^{\mathrm{percept}}+\lambda_{4}L_{t}^{\mathrm{style}}+\lambda_{5}L_{t}^{\mathrm{adv}}\right),$$

where the weight coefficients $\lambda_{1},\lambda_{2},\cdots ,\lambda_{5}$ are hyperparameters of the proposed inpainting model. They are set to 1, 3, 0.08, 150, and 0.2, respectively, under the guidance of validation set.

## 3. Experiments

In this section, we conduct extensive experiments and comparative studies to demonstrate the effectiveness and the superiority of the proposed inpainting model.

Source code is available at https://github.com/yabg-shuai666/Inpainting (accessed on 22 August 2021).

### 3.1. Experimental Setup

Three benchmark databases, namely Places2 [57], Paris StreetView [58], and CelebA [59], are commonly used in the image inpainting community. The Places2 database [57] contains more than 10 million images comprising over 400 indoor or outdoor scene categories. The Paris StreetView database [58] contains about 60 K panoramas scraped from Google Street View. Two perspective images have been carefully cropped from each panorama. These images mainly reflect building facades appearing in the modern city. The CelebA database [59] contains more than 200 K face images with large pose variations and background clutter. Images in these databases cover a variety of scenes and contents, allowing us to train an inpainting model more suitable for real-world applications.

We prepare the training set, the validation set, and the test set via the following steps. First, randomly select 50 K images from each database. Second, normalize their spatial resolutions to 256×256 through appropriate cropping and scaling operations. Third, artificially fabricate the corrupted images $I_{1}^{\mathrm{in}}$ according to the binary masks $M_{1}^{\mathrm{in}}$, where t=1 means the initial inpainting stage. In our experiments, we adopt the irregular binary masks prepared in [30]. Fourth, group the images into three sets: 600 images for testing, another 600 ones for validating, and the remaining ones for training.

Five image inpainting models [30,35,41,47,48], which are the representatives in specialized convolutions, contextual attention, and progressive strategies, serve as baselines for performing the comparative studies. Hereafter, these baselines are called PConv [30], Shift-net [35], MUSICAL [41], LSTM-PGN [47], and FRRN [48] for short. Unless explicitly stated, the total number of inpainting stages *T* is set to 4, 8, and 4, respectively, for LSTM-PGN [47], FRRN [48], and the proposed one. All of the image inpainting networks are trained by the Adam optimizer with default settings [65].

Our computing device is a workstation with a 3.20 GHz Intel Xeon W-2104 CPU and a 11GB NVIDIA GeForce RTX 2080Ti GPU. Our programming environment is PyTorch v1.2 installed on Ubuntu v18.04 operation system.

### 3.2. Qualitative Results

**Figure 4:**
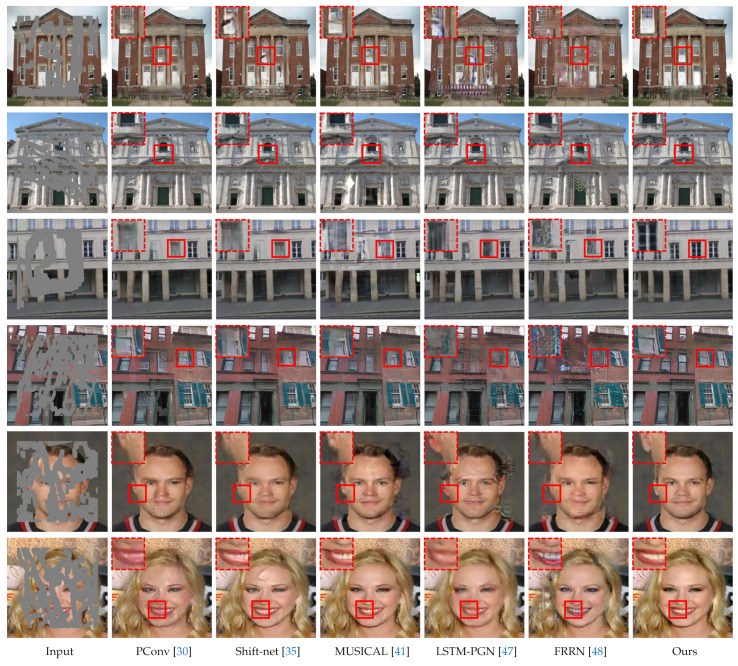
Qualitative results for visual comparisons. From top to bottom, the first two images come from Places2 [57], the middle two from Paris StreetView [58], and the last two from CelebA [59]. All images are free from post-processing.

Figure 4 exhibits the qualitative results. The first column of Figure 4 lists the corrupted images, which serve as the inputs to the inpainting networks. From top to bottom, the first two images come from the Place2 [57], the middle two from the Paris StreetView [58], and the last two from the CelebA [59] databases. The irregular gray region indicates the corrupted part, and the corresponding corruption rates are 30.63%, 25.00%, 31.49%, 41.89%, 39.27%, and 38.98%, respectively. The second to seventh columns of Figure 4 display the inpainted results, in which zoomed-in details are placed at the top-left corner.

As we can see, the PConv model [30] fails to suppress the blurring and upsampling artifacts. This may be partly due to the absence of the adversarial loss and partly due to the interference introduced by the fully spatial feature normalization. Although the shift-net [35] and the MUSICAL [41] models are equipped with the contextual attention modules, they still occasionally generate the distorted structures in the filled region. This implies that allocating attention within a single stage may synthesize wrong features to some extent. The LSTM-PGN [47] and FRRN [48] models tend to fill in the hole according to surrounding colors. For example, their resulting images in the first row show that most of the filled regions share the similar hue (red) with their surroundings. This verifies that these two models [47,48] can only perceive a part of the surroundings throughout all inpainting stages. By contrast, our model successfully generates semantically reasonable and visually realistic contents with clear textures and sharper details. These qualitative comparisons demonstrate the superiority of the proposed model.

### 3.3. Quantitative Results

Table 1 lists the quantitative results, in which four canonical metrics, i.e., SSIM (Structural Similarity), PSNR (Peak Signal-to-Noise Ratio), FID (Fréchet Inception Distance) [66], and $\ell_{1}$-norm, are used to objectively evaluate the inpainting quality. In this experiment, we consider three ranges of the corruption rates: 20–30%, 30–40%, and 40–50%, and correspondingly divide the test set into three groups, each of which comprises 150 test images. The values recorded in Table 1 are the average evaluation scores over 150 test images.

As we can see, in most cases, the proposed model achieves better evaluation scores than the baselines, especially on the CelebA database [59]. For the case of low corruption rate (20–30%), our SSIM (PSNR) scores reach 0.901 (25.53 dB), 0.915 (28.17 dB), and 0.941 (31.10 dB) on the three databases, and their average equals 0.919 (28.27 dB). For the case of middle corruption rate (30–40%), our average SSIM (PSNR) score is 0.868 (25.93 dB). These evaluation scores reflect that the proposed model can fill in the hole with visually pleasing contents even when 20–40% pixels are unknowns. For the case of the high corruption rate (40–50%), although the average SSIM (PSNR) score drops down to 0.77 (23.58 dB), the principal outlines in the filled region can still be recognizable. Additionally, the proposed model behaves better in terms of FID and $\ell_{1}$-norm, which jointly supports the qualitative comparisons.

Interestingly, we find that the MUSICAL model [41] usually achieves the second best performance, which just ranks below ours. This suggests that the multi-scale contextual attention mechanism is helpful for the image inpainting task, and equipping it with the progressive inpainting strategy (our main proposal) does further boost the performance.

### 3.4. Ablation Studies

In this section, we study how each part of the proposed model contributes to the inpainting performance from the following four perspectives: the MSCA module, the SE-based fusing module, the number of inpainting stages, and the collaborations between inpainting stages. Unless explicitly stated, the ablation studies are performed on the Places2 database [57] with the corruption rate of 30–40%.

#### 3.4.1. Ablation Study on the MSCA Module

Recall that $X_{n^{-}}$ and $Y_{n^{+}}$ are fed into the MSCA module. The former is the feature map at the nth-to-end layer of the shared encoder network, while the latter is nth feature map of the long-range attention branch. This ablation study is devoted to examining the influence of the position *n* on the inpainted results. As shown in Table 2, we consider six settings: n=⌀, n={4}, n={5}, n={4,5}, n={3,4}, and n={5,6}, where the null set *⌀* indicates that the MSCA module is turned off.

The scores in the column of n=⌀ are the worst, which demonstrates that the MSCA module are indeed useful for the image inpainting task. Moreover, we find that cascading two MSCA modules on the deeper layers usually outperforms the other settings. In this paper, the MSCA module is configured at the 4th and 5th layers of the long-range attention branch, as shown in Figure 1.

**Figure 5:**
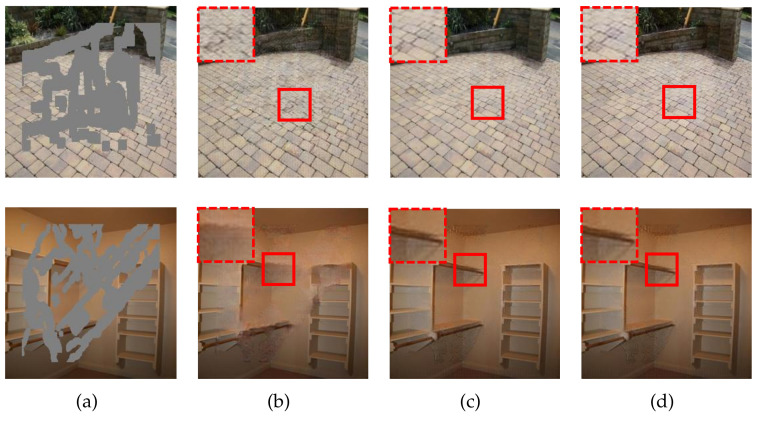
Qualitative results for the ablation study on the MSCA module. (**a**) Input images. (**b**) Resulting images for the setting n=⌀. (**c**) Resulting images for the setting n={4,5}. (**d**) Resulting images for the setting n={3,4}.

In Figure 5, we provide the qualitative results under the settings: n=⌀, n={4,5}, and n={3,4}. Without the MSCA module, the predictions in the zoomed-in box look rather blurry and suffer from texture distortions. By contrast, the inpainted results in the third and fourth columns look clearer and sharper. Especially for the second example, the principal content in the shelf area has been restored successfully. These observations are consistent with the scores in Table 2.

#### 3.4.2. Ablation Study on the SE-Based Fusing Module

The SE-based fusing module is to refine the local and long-range features. To verify its effectiveness, in this ablation study, we consider three reweighting modes: SE, random, and uniform. The SE mode, as shown in Figure 3, means that the reweighting coefficients are generated from the learnable fully connected layers. In the random mode, the reweighting coefficients are sampled from a random distribution. In the uniform mode, the reweighting coefficients are fixed to 1/256. For convenience, all testing images are corrupted by five irregular binary masks, in which the corruption rates are 20.3%, 32.9%, 35.9%, 42.2%, and 45.0%.

**Figure 6:**
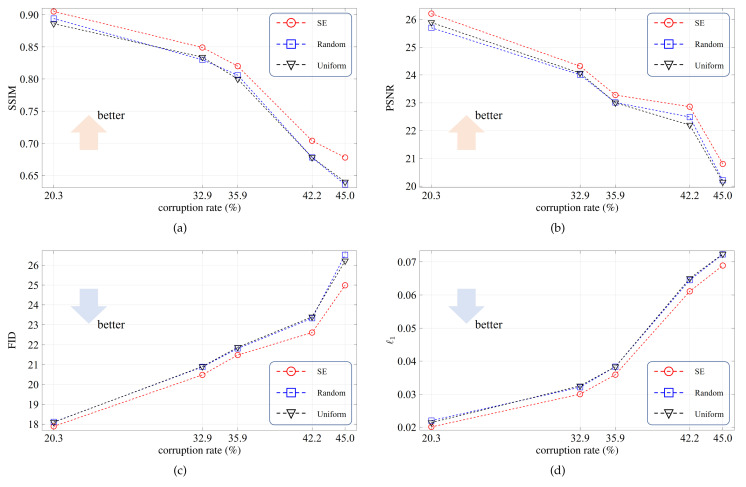
Quantitative results for the ablation study on the SE-based fusing module. red(**a**) The SSIM scores. (**b**) The PSNR scores. (**c**) The FID scores. (**d**) The $\ell_{1}$ scores.

**Figure 7:**
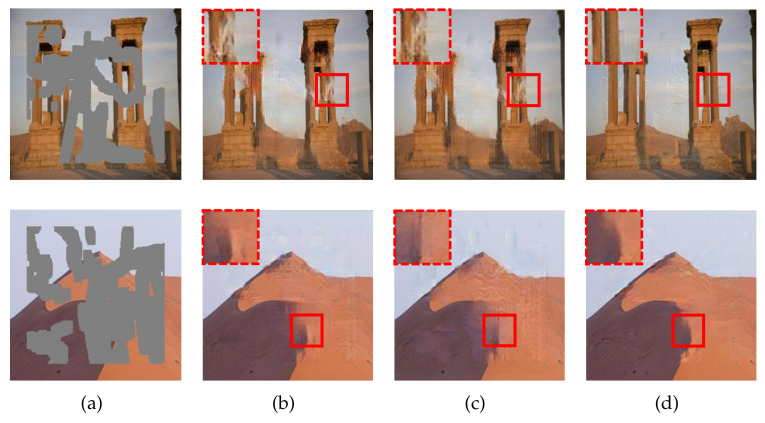
Qualitative results for the ablation study on the SE-based fusing module. The corruption rates, from top to bottom, are 32.9% and 42.2%, respectively. (**a**) Input images. (**b**) Resulting images for the random mode. (**c**) Resulting images for the uniform mode. (**d**) Resulting images for the SE mode.

The average evaluation scores are plotted in Figure 6. As we can see, the SE mode outperforms the other two modes by clear margins, and the superiority becomes more significant for larger corruption rates. Figure 7 exhibits the resulting images for the three modes. For the first example, the SE mode completely reconstructs the pillar area while the other two modes suffer defeat. These results demonstrate that the SE-based fusing module plays a key role in comprehensively refining the two feature flows.

#### 3.4.3. Ablation Study on the Number of Inpainting Stages

The total number of inpainting stages *T* highly affects the final inpainting performance. In this ablation study, we experimentally investigate what is the appropriate value of *T*. To this end, we set *T* to 1, 4, and 6, respectively, in the course of training. Table 3 records the evaluation scores. As expected, multiple inpainting stages, i.e., T=4 or 6, is superior to the single inpainting stage, i.e., T=1. Comparing the last two columns of Table 3, we find that the quality gain is tiny when increasing *T* from 4 to 6. Based on our measurement, this tiny quality gain, however, consumes an additional 6.9G FLOPs (Floating Point Operations). In order to strike the balance between the inpainting quality and the computational cost, we recommend setting *T* to 4.

**Figure 8:**
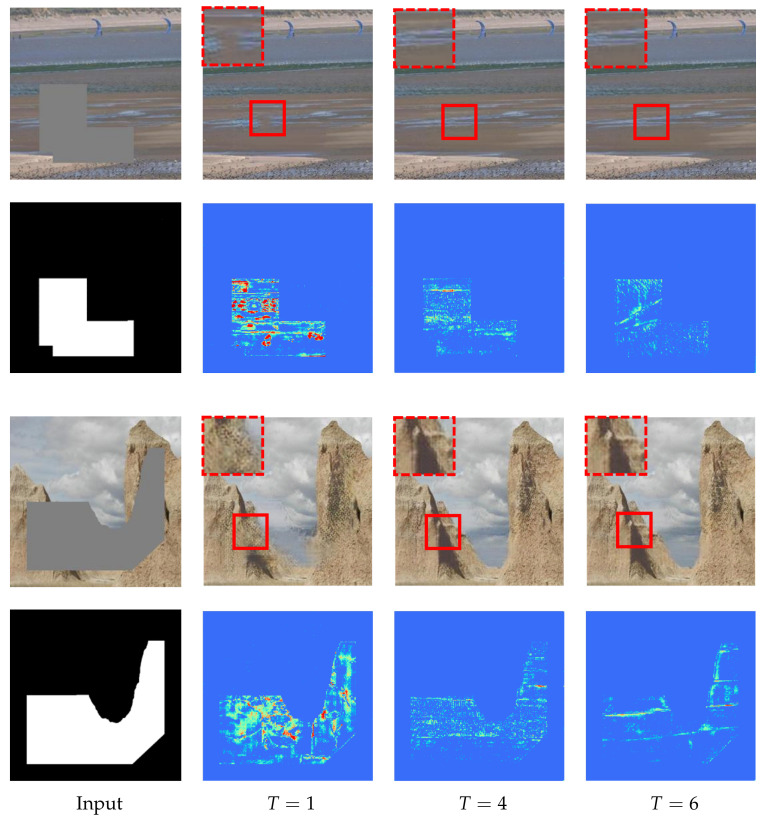
Qualitative results for the ablation study on the number of inpainting stages. The corruption rates, from top to bottom, are 23.1% and 33.7%, respectively.

The first and third rows of Figure 8 show the resulting images, from which we find that more inpainting stages help to restore the realistic boundaries between objects. The second and fourth rows of Figure 8 visualize the feature deviations, which are obtained at the layer after the fusing module (the pink one in Figure 1) by calculating the difference between the feature maps of the input and groundtruth images. We focus on the filled region and highlight larger feature deviations in hot colors. As we can see, the second column of Figure 8 (i.e., T=1) contains more noticeable hot spots than the other columns. These results suggest that the designed progressive inpainting strategy is useful for the image inpainting task to reduce the feature deviations and narrow the semantic gap.

#### 3.4.4. Ablation Study on the Collaborative Effect between Inpainting Stages

As discussed before, the filled region at the tth stage is regarded as the valid region at the (t+1)th stage. In other words, the MSCA module at the (t+1)th stage treats the filled region as the new source for synthesizing the inpainting-oriented features. In this ablation study, we attempt to reveal the collaborative effect between inpainting stages through two trials.

**Figure 9:**
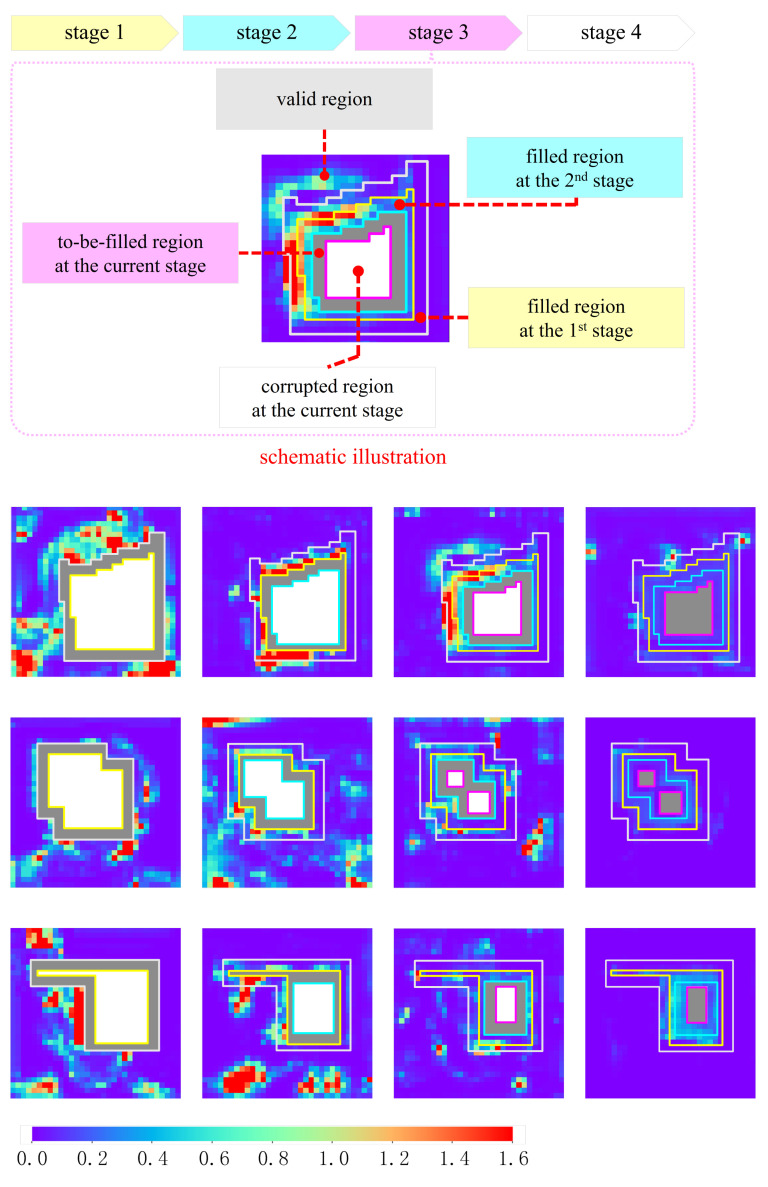
Heat maps of the attention scores. A schematic illustration is shown at the top-half panel, in which four inpainting stages are demarcated by closed-loop frames depicted in colors. Three rows at the bottom-half panel exhibit the heat maps for three practical examples. Four columns, from left to right, correspond to the first, the second, the third and the fourth inpainting stages, respectively.

In the first trial, we visualize attention scores, which reflect how the patches in the to-be-filled region refer to the valid region. The actual calculation is the complement to the one shown in Figure 2 because the to-be-filled patches, rather than the valid patches, serve as the kernels in this trial. For simplicity, we focus only on the second MSCA module, namely the one configured at the 5th layer of the long-range attention branch. Figure 9 shows the visualized heat map, which is obtained by summing the attention scores over the channel dimension. In the heat map, the hot and cool colors represent the high and low attention scores, respectively.

For the first inpainting stage, only the valid region is the source for synthesizing features, and all of the valid patches are likely to contribute to the inpainting task in a learnable way. For the other inpainting stages, the MSCA module borrows the features not only from the valid region but also from the filled ones. As we see in the first example, more hot colors are accumulated in the filled regions. This demonstrates the existence of the collaborative effect between inpainting stages.

**Figure 10:**
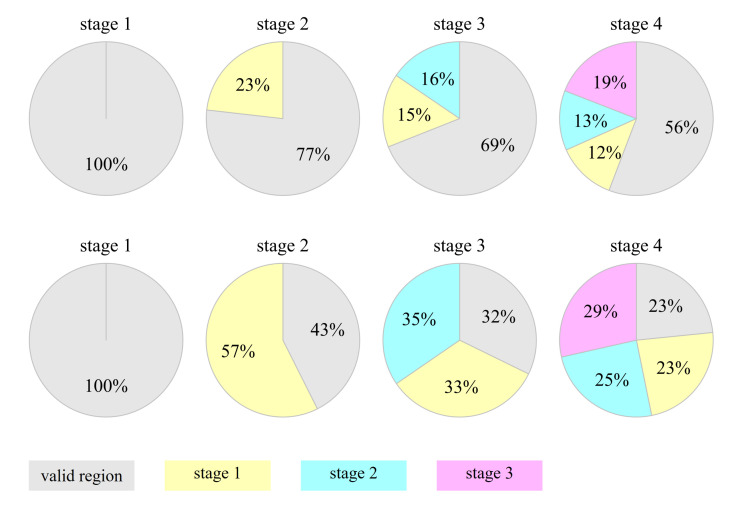
Region-wise contribution at each inpainting stage. The second row corresponds to the area-normalized contribution.

Figure 10 further shows how different regions contribute to the inpainting task at each stage. The contribution of a region is defined as the proportion of attention scores received by that region. From the first row of Figure 10, we see that, except for the first stage, all of the filled regions contribute to the inpainting task. Especially at the 4th inpainting stage, the filled regions receive nearly half of the attention scores. Intuitively, the larger the region, the higher probability to receive the attention scores. For a fair comparison, we count the area-normalized contribution by using the attention score per unit area. From the second row of Figure 10, we see that, except for the first stage, each filled region roughly makes the same contribution the valid region. These statistical results demonstrate the usefulness of the collaborative effect between inpainting stages.

**Figure 11:**
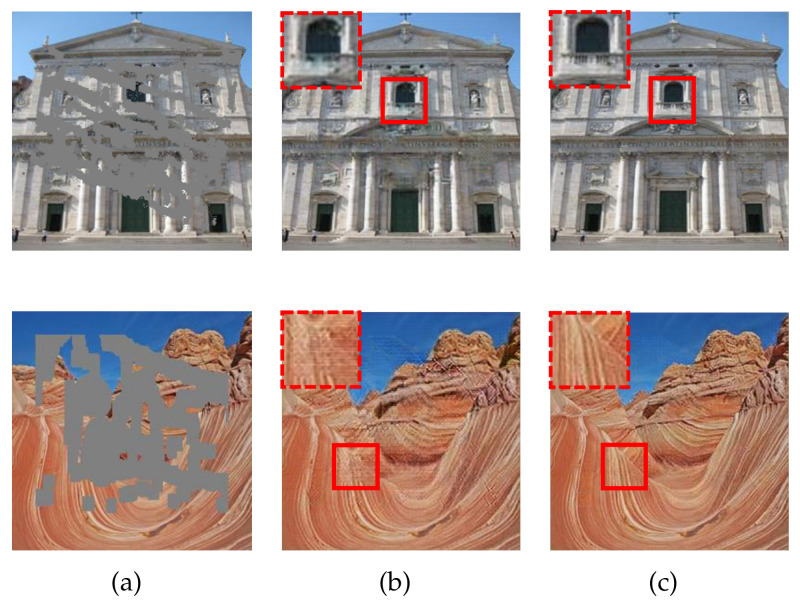
Qualitative results for the ablation study on the collaborative effect between inpainting stages. The corruption rates, from top to bottom, are 25.0% and 38.4%, respectively. (**a**) Input images. (**b**) Resulting images when the filled regions are switched off. (**c**) Resulting images when the filled regions are switched on.

In the second trial, we deliberately exclude the filled regions from the MSCA module. In doing so, only the valid region is available for the MSCA module to synthesize the features, regardless of the inpainting stage. In Figure 11, we show the resulting images for qualitative comparisons. As we see, the resulting images in Figure 11b contain observable upsampling artifacts and content deviations. For the top example, some white spots improperly appear in the black background. See the zoomed-in box for details. The reason for this is as follows. In this example, white is the dominant color in the valid region. When the filled regions are switched off, the MSCA module runs a higher risk of borrowing wrong features from the white region. By contrast, the resulting images in the last column have visually realistic and semantically plausible contents. This is because the filled regions extend the available source for synthesizing features to reduce the risk of allocating wrong attention. These results demonstrate the effectiveness of the collaborative effect between inpainting stages.

## 4. Conclusions

In this paper, we propose progressively inpainting the corrupted images based on a shared encoder network and a forked-then-fused decoder network. We design a PC-RN unit, which can perceive the valid information whilst suppressing the incompleteness-caused interference. The proposed decoder network forks into the local reception branch and the long-range attention branch (with two MSCA modules) at the beginning, and the two feature flows are adaptively refined through a SE-based fusing module. The progressive inpainting strategy has the collaborative effect in the sense that the filled region at the previous stage helps the MSCA module find matching features. We evaluate our inpainting model on three benchmark databases [57,58,59] and conduct the extensive comparative studies and ablation studies. Experimental results demonstrate the effectiveness and the superiority of the proposed model compared with the state-of-the-art works [30,35,41,47,48]. Four ablation studies reveal the functionality of each module for the inpainting task.

## Figures and Tables

**Table 1:** Quantitative results for numerical comparisons. The arrow “↑” (or “↓”) is intended to indicate that a higher (or lower) value is better. The best and the second best scores are highlighted by bold and underline, respectively.

Database		Places2			Paris StreetView			CelebA		
**Corruption Rate**		**20–30%**	**30–40%**	**40–50%**	**20–30%**	**30–40%**	**40–50%**	**20–30%**	**30–40%**	**40–50%**
SSIM (↑)	PConv	0.883	0.790	0.659	0.908	0.860	0.767	0.928	0.892	0.827
	Shift-net	0.887	0.790	0.667	**0.916**	0.870	0.774	0.930	0.910	0.827
	MUSICAL	0.899	0.795	**0.678**	0.913	0.872	0.779	0.939	0.924	0.849
	LSTM-PGN	0.895	0.799	0.672	0.909	0.866	0.774	0.935	0.915	0.837
	FRRN	0.892	0.800	0.670	0.910	0.867	0.775	0.937	0.920	0.851
	Ours	**0.901**	**0.803**	**0.678**	0.915	**0.875**	**0.783**	**0.941**	**0.926**	**0.853**
PSNR (↑)	PConv	25.00	22.33	19.99	27.99	24.40	22.72	30.45	27.10	22.93
	Shift-net	25.48	22.79	19.92	28.09	25.15	22.90	30.78	27.32	23.01
	MUSICAL	25.01	23.04	21.43	28.15	26.03	**24.69**	31.06	27.98	24.39
	LSTM-PGN	25.32	22.46	19.86	28.07	25.67	23.07	30.89	27.78	23.29
	FRRN	25.28	22.98	20.56	28.10	25.90	24.11	**31.10**	28.00	24.00
	Ours	**25.53**	**23.62**	**21.49**	**28.17**	**26.09**	24.67	**31.10**	**28.09**	**24.58**
FID (↓)	PConv	18.31	23.02	25.43	20.11	22.13	27.00	18.06	18.98	25.02
	Shift-net	18.76	22.78	24.78	18.66	20.97	26.03	18.01	19.03	24.87
	MUSICAL	18.10	21.27	**23.69**	18.66	20.78	23.56	17.03	**18.26**	22.22
	LSTM-PGN	18.98	22.30	25.12	19.92	22.09	25.84	17.94	18.90	24.95
	FRRN	18.34	22.00	24.98	19.27	21.62	24.35	17.26	18.45	23.41
	Ours	**18.02**	**21.19**	23.76	**18.64**	**20.76**	**23.36**	**16.99**	**18.26**	**22.10**
$\ell_{1}$ (↓)	PConv	0.0278	0.0483	0.0811	0.0201	0.0344	0.0587	0.0159	0.0249	0.0411
	Shift-net	0.0261	0.0430	0.0735	**0.0180**	0.0332	0.0559	0.0165	0.0250	0.0410
	MUSICAL	0.0226	**0.0319**	**0.0666**	0.0184	**0.0268**	0.0478	0.0143	0.0201	0.0345
	LSTM-PGN	0.0260	0.0352	0.0765	0.0192	0.0302	0.0578	0.0159	0.0252	0.0419
	FRRN	0.0256	0.0340	0.0690	0.0192	0.0289	0.0499	0.0152	0.0221	0.0403
	Ours	**0.0223**	**0.0319**	0.0669	**0.0180**	**0.0268**	**0.0473**	**0.0141**	**0.0198**	**0.0342**

**Table 2:** Quantitative results for the ablation study on the MSCA module.

	Position *n*		⌀	{4}	{5}	{4, 5}	{3, 4}	{5, 6}
	SSIM (↑)		0.788	0.796	0.800	**0.803**	**0.803**	0.800
	PSNR (↑)		23.37	23.53	23.58	**23.62**	23.61	23.59
	FID (↓)		21.50	21.27	21.25	21.20	**21.19**	21.25
	$\ell_{1}$ (↓)		0.0344	0.0330	0.0322	**0.0318**	0.0319	0.0323

**Table 3:** Quantitative results for the ablation study on the number of inpainting stages.

*T*	1	4	6
SSIM (↑)	0.798	0.803	**0.800**
PSNR (↑)	22.99	**23.62**	**23.62**
FID (↓)	22.07	21.19	**21.18**
$\ell_{1}$ (↓)	0.0341	**0.0318**	0.0320

## Data Availability

Data sharing not applicable.
